# Complementary Interventions Using Technology for Individuals With Infertility Using Assisted Reproductive Technology: A Scoping Review

**DOI:** 10.1111/nhs.70227

**Published:** 2025-09-05

**Authors:** Jiwon Lee, Jaejin Kang, Jo Woon Seok

**Affiliations:** ^1^ School of Nursing Dongnam Health University Suwon South Korea; ^2^ College of Nursing, The Catholic University of Korea Seoul South Korea; ^3^ College of Nursing, Ewha Womans University Seoul South Korea

**Keywords:** assisted reproductive technology, complementary intervention, digital health, eHealth, infertility

## Abstract

The global rise in infertility highlights the need for personalized informational and psychological support. Digital health technology offers a promising avenue; however, knowledge gaps remain regarding optimal delivery methods, outcomes, and limitations. This scoping review synthesizes recent evidence on complementary interventions using technology for individuals with infertility undergoing reproductive technology‐assisted interventions and evaluates their effectiveness and limitations. Seven databases were systematically searched to identify studies published in English or Korean between 2000 and December 2023. The risk of bias was assessed using the Joanna Briggs Institute checklist, and data were synthesized using a standardized template. Of 1353 screened studies, 16 were selected, featuring interventions such as web‐based programs, mobile applications, and online meetings. Positive outcomes were observed in knowledge, stress reduction, self‐efficacy, coping, and dietary behaviors. However, mixed results were found for fertility‐related stress, depression, and anxiety, with no significant improvements in fertility‐related quality of life, health behaviors, and pregnancy rates. While digital interventions show promise, further research is needed to validate their effectiveness through large‐scale randomized controlled trials and to develop standardized assessment tools for better implementation and evaluation.

**Summary**

- The studies employed various digital tools (e.g., webbased platforms, mobile applications, online meetings, email and text messaging, and online communities) to deliver educational, psychological, and cognitive behavioral interventions.
- The review indicates that complementary interventions using technology (CITs) for individuals with infertility can lead to beneficial outcomes in stress reduction, self‐efficacy, and coping behaviors. However, the evidence for improvements in fertility‐specific outcomes (e.g., fertility‐related stress, fertility‐related quality of life, and pregnancy rates) remains inconclusive.
- Currently, there is limited evidence that CITs affect the success rate of assisted reproductive technologies. Future research should focus on comprehensive interventions, validated scales, and biomarkers to strengthen evidence‐based practice.

## 1 Introduction

The World Health Organization (WHO) defines infertility as the inability to conceive after 1 year of unprotected intercourse. The prevalence of infertility is estimated to be approximately 12.9% worldwide, with a continuous upward trend over the years (Zhu et al. 2023). The burden of infertility among women is steadily increasing, leading to a rapid increase in the number of infertility treatments (Wang et al. 2024). Marriage and childbirth at later ages increase the likelihood of fertility decline, leading to an increase in the number of couples dealing with infertility (Owen et al. 2024). The causes of infertility include female, male, mixed, and unexplained factors. Female factors, accounting for approximately half of infertility cases, include uterine factors, menstrual and ovulation disorders, and ovarian disorders (Obeagu et al. 2023). In addition, modifiable lifestyle factors, such as high‐fat diets, delayed childbearing, smoking, alcohol abuse, sexual behavior, anxiety/depression, and personal perceptions and beliefs, have been shown to negatively impact fertility (Emokpae and Brown 2021).

Despite the growing demand for informational support for infertility, available information and support remain insufficient. In addition, women with infertility often suffer from anxiety, stress, and depression due to physical and psychological distress, relationships with spouses, the burden of pregnancy, loneliness, low self‐esteem, and lack of social recognition; this is considered a public health concern (Braverman et al. 2024). In particular, women undergoing assisted reproductive technology (ART) interventions experience high anxiety, stress, and low quality of life due to uncertainties about procedural outcomes, the duration required for conception, and physical burdens such as ovarian hyperstimulation, egg retrieval, and potential side effects (Koert et al. 2021). Therefore, the unmet needs of individuals with infertility with regard to comprehensive guidance on diagnostic tests and ARTs to help them make informed family planning decisions should be addressed and satisfied, along with continuous counseling and management to support their psychological burdens (Owen et al. 2024).

Complementary interventions are additional interventions such as nutritional supplements, psychological therapies, mind and body practices, and physical therapies provided by healthcare professionals (Ng et al. 2023). Face‐to‐face complementary interventions for individuals with infertility include lifestyle modifications such as diet and exercise. In addition, psychological interventions (e.g., counseling, relaxation, cognitive behavioral therapy, and mindfulness) effectively reduce depression/anxiety and stress and improve infertility‐related quality of life (Untaaveesup et al. 2024).

Digital health, such as wearable devices and health information technology, effectively enhances the personalization of healthcare, enabling more tailored and patient‐specific care approaches. Previous studies applying digital interventions provided infertility‐related knowledge, psychological support, shared pregnancy success experiences, and activated distance counseling with medical experts (Sykes et al. 2020).

However, there are no comprehensive reviews of the specific methods and outcomes of complementary interventions using technology (CITs) for individuals with infertility. Therefore, the purpose of this scoping review is to identify and synthesize existing literature on CITs provided to individuals with infertility, particularly those undergoing ART. The goal is to evaluate the methods, effectiveness, and limitations of these interventions and to provide implications for future research and practical implementation. To this end, this scoping review addresses the following research questions:

1. What types of CITs have been studied for individuals with infertility using ART?
2. What digital health tools, outcome measures, and intervention durations have been used in CITs for individuals with infertility, and what effects have been reported?
3. What are the key barriers and considerations for effectively implementing CITs for individuals with infertility in community settings?

## 2 Design

This review was conducted in accordance with the Joanna Briggs Institute methodology for Scoping Reviews (Peters et al. 2020) and reported following PRISMA‐ScR (Preferred Reporting Items for Systematic reviews and Meta‐Analyses extension for Scoping Reviews) checklist (Tricco et al. 2018). The protocol outlining the approach for this scoping review was registered in the Open Science Framework (OSF) registries at https://doi.org/10.17605/OSF.IO/4NKZG.

## 3 Materials and Methods

### 3.1 Eligibility Criteria

The inclusion criteria for this review were based on the principles of population, concept, and context. The population or participants were defined as couples with infertility. The concept was defined as receiving complementary or non‐pharmacological interventions. The context required the use of technology in delivering the intervention. In addition, we included articles published since 2000, as the widespread adoption of the internet began in the early 2000s. We excluded conference proceedings, review articles, and study protocols, as they did not report original research findings suitable for this review. The detailed inclusion and exclusion criteria are as follows:

### 3.2 Inclusion Criteria

1. Couples with infertility (either female or male) who were planning to undergo or were undergoing ARTs (e.g., in vitro fertilization [IVF], intracytoplasmic sperm injection [ICSI], and artificial insemination).
2. Individuals who were not already pregnant.
3. Received complementary interventions (e.g., informational, psychosocial, or physical), rather than pharmacological interventions.
4. Use of technology integrated with Information and Communication Technology (ICT).
5. Written in either English or Korean.

### 3.3 Exclusion Criteria

1. Individuals diagnosed with malignancies who had undergone or were undergoing chemotherapy or radiotherapy.
2. Individuals diagnosed with psychiatric disorders.
3. Received interventions related to contraception, birth control, or fertility preservation.
4. Studies on the effectiveness of pharmacological interventions.
5. Review articles, magazines, webpages, books, letters, poster presentations, and editorials.
6. Full text not available.

### 3.4 Type of Database and Search Term

All accessible databases, including PubMed, CINAHL, Ovid‐Medline, Cochrane Library, Scopus, Embase, and PsycINFO, were searched for eligible studies up to December 2023. In addition, experts were consulted to help identify search terms most appropriate for the scope of this review. The search was not limited by publication status or date. Detailed search strategies, including the specific terms used, are provided in the Supporting Information.

### 3.5 Screening and Selection of Eligible Studies

**FIGURE 1:**
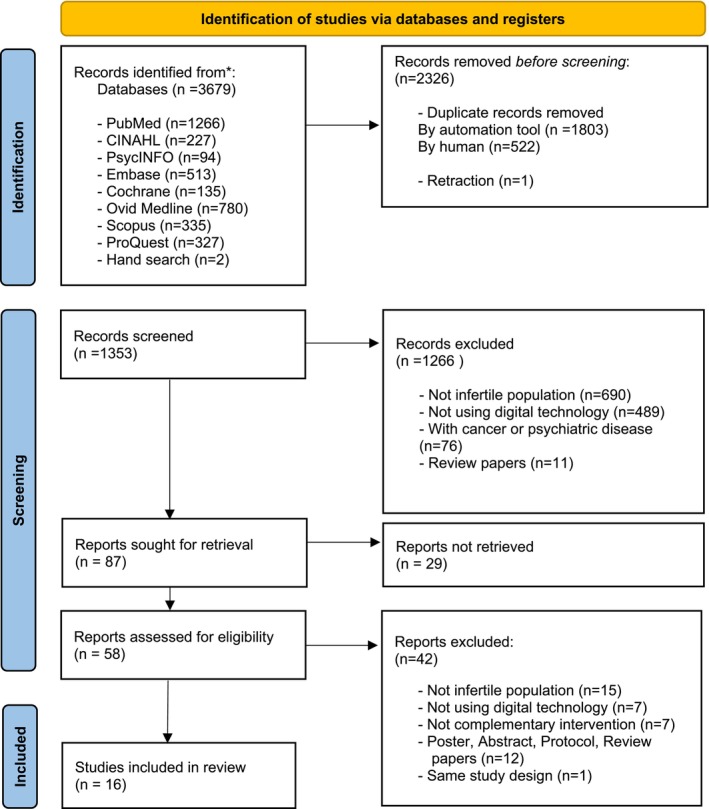
PRISMA flow diagram for article selection.

All searched studies were extracted using the EndNote 20 management software program (Clarivate Analytics, Philadelphia, PA, USA), and duplicates were automatically removed. Subsequently, two researchers (J.K. and J.W.S.) independently screened the titles and abstracts according to the inclusion and exclusion criteria. Any disagreements were resolved through consensus with another researcher (J.L.), who also re‐checked the literature. We reviewed the full texts, re‐screened the included studies, and selected the relevant literature for further analysis. The reasons for the exclusion of studies are described in the PRISMA flow chart (Figure 1). Ultimately, the review included eight randomized controlled trials (RCTs) (Vause et al. 2018; van Dongen et al. 2016; Njogu et al. 2023; Oostingh et al. 2020; Timmers et al. 2021; Sexton et al. 2010; Boedt et al. 2023; Cousineau et al. 2008), four cross‐sectional studies (Grunberg et al. 2020; Jones et al. 2020; Madeira et al. 2018; Langarizadeh et al. 2022), three quasi‐experimental studies (Hojeij et al. 2023; Sparidaens et al. 2023; Cercato et al. 2022), one mixed‐method study (Cercato et al. 2022), and one qualitative study (Aarts et al. 2015).

### 3.6 Data Extraction and Synthesis

The final eligible studies were decided upon, and information for narrative synthesis was extracted, including the following components: author, published year, country, primary aim of the study, design, participants (age, sample size, duration of infertility, and treatment), key findings, and study limitations.

To synthesize detailed information related to CITs, we incorporated a template for intervention description and replication (TIDieR) based on previous studies (Backman et al. 2023; Hoffmann et al. 2014). This included the mode of digital health intervention, why (purpose of the study), what (content of the intervention), who (personnel who delivered the intervention), how (delivery mode or used technology), when, and how much (time and duration of intervention), and tailoring (whether it was an individualized and customized intervention).

To avoid discrepancies, an author extracted the data, followed by confirmation by another author.

### 3.7 Risk of Bias Appraisal

Risk of bias was appraised using the JBI Critical Appraisal Checklist (JBI, n.d.), following the approach outlined in a previous report (Barker et al. 2023). Two researchers (J.L. and J.W.S.) independently conducted the assessments, and any disagreements were resolved through discussion with a third researcher (J.K.). The purpose of this scoping review was to determine the breadth of existing literature on CITs for individuals with infertility. Accordingly, all eligible evidence was considered and included in the synthesis. The risk of bias in individual studies was categorized based on the proportion of checklist items rated “yes,” using criteria adapted from a previous study (Goplen et al. 2019): low risk (≥ 70% “yes”), moderate risk (50%–69% “yes”), and high risk (< 50% “yes”).

### 3.8 Ethical Consideration

This study did not collect any personal, confidential, or sensitive information on individuals; it only utilized existing literature. Thus, ethical approval from the institutional review board was not necessary.

## 4 Results

### 4.1 Characteristics of Included Studies

The characteristics of the included studies are summarized in Table 1. Eight studies were conducted in Europe, while the others were conducted in North America (*n* = 6), Asia (*n* = 1), and Africa (*n* = 1). The study designs included RCTs (*n* = 8) and quasi‐experimental (*n* = 2), cross‐sectional (*n* = 4), mixed‐method (*n* = 1), and qualitative (*n* = 1) studies. The participants in these studies varied and included patients diagnosed with infertility as well as individuals who were planning to undergo or were undergoing ART interventions. Nine studies (56.3%) focused solely on women, four on couples, and three on both men and women. The average age of participants in these studies ranged from 31.5 to 39.3 years. In the quantitative studies, the sample size ranged from 18 to 3097 participants, whereas the qualitative study had seven participants. The risk of bias assessment identified nine studies with low risk, seven with moderate risk, and one with high risk. Notably, one study (Cercato et al. 2022) employed both quasi‐experimental and qualitative methods and was assessed using two tools. Thus, while 16 studies were included, the total number of risk of bias assessments was 17 (Table 1).

**TABLE 1:** Characteristics of included studies.

No.	First author, country (year)	Design	Participants	Sample size	Age (mean)	Digital health tool name (explanation)	Measures	Key findings	Limitation	Risk of bias^a^
1	Vause, Canada (2018)	RCT	– Women – Undergoing first cycle of IVF	IG: 21 CG: 19	IG: 34.3 CG: 32.9	Web IVF education session (web‐based teaching tool)	– IVF knowledge questionnaire – 10‐item Perceived stress scale – Satisfaction survey	– Knowledge and stress↑ (pre vs. post) – Satisfaction ↑ (IG vs. CG)	– Generalizability (no standardized web‐based IVF teaching protocol) – Selection bias (motivated participants)	11/13 (84.6%) Low risk
2	Grunberg, Canada (2020)	Cross‐sectional	– Men and women – Have history of infertility	18	39.3	Online peer support (online infertility peer supporter training program)	– Experiences questionnaire – Training materials questionnaire	– High program satisfaction – Helpful training materials	– Technological issues – Peer supporters doubted their effectiveness or ability—Scheduling conflicts – Unable to recruit many men	5/8 (62.5%) Moderate risk
3	Cercato, Italy (2022)	Quasi‐experimental and qualitative study	– Women – Experienced or undergoing ART	22 Interview: 15	36 (median)	PSYDIT platform (web‐based psychological support platform)	– Feasibility and utility items – Group behavior evaluation (by psychologists)	– High feasibility and utility – Positive therapeutic benefits (peer support)	– Participant control (inconsistency of participation, disparity in the time from the diagnosis) – Need for improvement in program (digital pathway)	#1 4/9 (44.4%) High risk #2 5/10 (50.0%) Moderate risk
4	Van Dongen, Netherlands (2016)	RCT	– Women – Intention to start ART	IG: 23 CG: 34	IG: 32.0 CG: 32.4	Digicoach (web‐based personalized psychoeducation and CBT program)	– Hospital anxiety and depression scale	– Anxiety and depression↓ (IG vs. CG)	– Generalizability (30% dropout, single center) – Need for improvement in program (timing of the protocol to the women's needs and preferences) – Need future evaluation (barriers and facilitators for participation)	10/13 (76.9%) Low risk
5	Hojeij, Netherlands (2023)	Quasi‐experimental Three group pre/post study	– Women – Attempting pregnancy	IG: 553 CG: 283 NG: 396	32 (median)	Smarter pregnancy coaching program (email and text message‐based personalized coaching on nutrition and lifestyle behavior)	– Dietary questionnaire – Attitude questions	– Vegetable intake and attitudes↑ (IG vs. CG) – Fruit intake and attitudes (n.s.) – Smoking behavior (n.s.)	– Absence of a control group (naturally conceiving women) – Risk of recall bias (self‐administered dietary assessment, self‐reported BMI) – Need objective measurements (biomarker, scale based on knowledge‐attitude‐practice model)	7/9 (77.8%) Low risk
6	Njogu, Kenya (2023)	RCT	– Women – Undergoing fertility treatment	SCV: 65 SC‐DS: 67 CG: 68	31.5	Self‐compassion‐based program (video and online meeting‐based self‐compassion program)	– Zung Self‐Rating Depression Scale – Zung Self‐Rating Anxiety Scale – Self‐Compassion Scale Short‐Form – Infertility Self‐Efficacy Scale Short‐Form – Pregnancy assessment	– Depression and anxiety↓ (SCV, SC‐DS vs. CG) – Self‐compassion and self‐efficacy↑ (SCV, SC‐DS vs. CG) – Pregnancy rates (n.s.)	– Generalizability (internet access participant only, narrow participant characteristics) – Need to expand population (men, fertility problems without seeking treatment) – Need future evaluation (participants' interaction with study materials) – Intervention should be conducted by trained healthcare professionals	11/13 (84.6%) Low risk
7	Jones, Canada (2020)	Cross‐sectional	– Men and women – Diagnosed with infertility	106	34.3	(Online fertility educational material on Clinic website)	– Usefulness of the online educational material	– Educational content was helpful in making fertility decisions	– Risk of recall bias (self‐reporting survey)	4/8 (50.0%) Moderate risk
8	Oostingh, Netherlands (2020)	RCT	– Couples – Intention to start IVF/ICSI within 3 months	IG: 401 CG: 420	IG: 33.5 CG: 33.5	Smarter pregnancy coaching program (email and text message‐based personalized coaching on nutrition and lifestyle behavior)	– Dietary risk score – Lifestyle risk score	– Nutrition behaviors↑(IG vs. CG) – Smoking and alcohol (n.s.) – Pregnancy rates (n.s.)	– Fail to achieve estimated sample size (300/500) – Generalizability (highly educated, exclude non‐Dutch speakers) – Risk of recall bias (self‐administered questionnaires) – Need future evaluation (effects of improvement of inadequate nutritional and lifestyle behaviors on pregnancy outcomes)	11/13 (84.6%) Low risk
9	Timmers, Netherlands (2021)	RCT	– Women – Scheduled for IVF or ICSI	IG: 22 CG: 16	IG: 32.4 CG: 32.8	Patient Journey App (application for educational material with personalized timing)	– Satisfaction with the information – Level of knowledge	– Satisfaction and knowledge↑ at 2 days after IVF medication intake (IG vs. CG)	– Generalizability (small sample size) – Need for improvement in program (involve patient input on contents, format, timing of push notifications) – Risk of recall bias (non‐validated self‐reported questionnaires) – Need to develop more personalized app (patient's anxiety or depression, stages of the treatment) – Need to expand population (other phases of the IVF, other treatments)	7/13 (53.8%) Moderate risk
10	Madeira, United states (2018)	Cross‐sectional	– Couples – Viewed the multimedia platform before IVF/OI‐IUI	IVF: 2046 OI‐IUI: 1051 Medical providers: 41	Not described	Engaged MD program (multi‐media platform with e‐learning system)	– 17 Item survey questionnaires (for patients) – Survey questionnaires (for medical providers)	– Helpful for consultations – Helpful to prepare for consent documents – Patients' understanding↑ [provider] – Saved up to 1 h per patient for education [provider]	– Participant control (patient experiences of using multimedia platform, baseline knowledge assessments) – Lack of a uniform implementation protocol (before vs. after physician consent conversation) – Need future evaluation (difference by age, effective timing, and place for interventions)	7/8 (87.5%) Low risk
11	Sexton, United states (2010)	RCT	– Women – Receiving infertility assessments and/or treatments	IG: 15 CG: 16	32.6	WCWI (web‐based intervention for coping with infertility)	– Revised Symptom Checklist 90 – Fertility Problem Inventory	– General stress ↓ (IG vs. CG) – Fertility‐related stress (n.s.)	– Generalizability (high socioeconomic status, small sample size) – Portion of the coping with infertility package is used – Need future evaluation (psychological needs of men, co‐assist of partners in coping with stress, how the materials are used by participants, investigating the full program)	10/13 (76.9%) Low risk
12	Boedt, Belgium (2023)	RCT	– Couples – Starting a first IVF	IG: 106 couples CG: 105 couples	IG: 32.3 CG: 32.4	PreLiFe‐program (mobile‐based tailored advice and skills training on diet, physical activity and mindfulness application with text messages and telephone interaction with a health care professional)	– Core Outcome Measures for Infertility Trials – Food Frequency Questionnaire – International Physical Activity Questionnaire – Depression, Anxiety and Stress Scale Short Form – Fertility‐Related Quality of Life Tool	– Cumulative ongoing pregnancy (n.s.) – Diet quality, fruit, and vegetable intake↑ (IG vs. CG) – Physical activity, emotional distress, fertility‐related quality of life (n.s.)	– Generalizability (high physical activity level, low overweight and obesity, Caucasian, highly educated, COVID‐19 pandemic limited its power and the actual use of mobile application) – Risk of recall bias (self‐reported lifestyle outcomes) – Need future evaluation (preconception lifestyle programs, effective means of encouraging a healthy lifestyle)	9/13 (69.2%) Moderate risk
13	Sparidaens, Netherlands (2023)	Quasi‐experimental Two group post only study	– Couples – Possible ICSI with surgical sperm retrieval	User: 35 couples Nonuser: 107 couples	User: 34.5 Nonuser: 34.5	myFertiCare (personalized information and interactive functionalities online application)	– User questionnaire based on human, organization and technology framework	– Good system usability – High user satisfaction – Knowledge about fertility treatment↑ – Coping with treatment↑	– Generalizability (low response rate 25%) – Risk of recall bias (self‐reported differences before and after app use) – Need to develop an app that also provides a benefit for the treatment team (making it easier or more efficient to provide care to patients or by making that care better)	5/9 (55.6%) Moderate risk
14	Cousineau, United states (2008)	RCT Solomon‐four group design	– Women – Diagnosed with infertility	IG: 49 IG′: 47 CG: 49 CG′: 43^b^	IG: 34.5 IG′: 34.3 CG: 34.1 CG′: 33.9	Infertility source (web‐based education and support program)	– Fertility problem inventory – Infertility self‐efficacy scale – Perceived negative support scale – Decisional conflict scale – Satisfaction questionnaire	– Global stress and infertility distress↓ (IG vs. EG) – Infertility self‐efficacy (IG vs. EG) – Helpful in making a medical decision (n.s.) – Informative and helpful (descriptive)	– Generalizability (actively seeking medical care participants) – Risk of recall bias (self‐report data) – Short follow‐up duration period (limit the use of biological outcomes such as pregnancy attainment) – Need to expand population (male partners, couple interactions, personal belief systems)	10/13 (76.9%) Low risk
15	Aarts, Netherlands (2015)	Qualitative	– Men and women – Receiving infertility assessments and/or first IUI/OI	7	Not described	(Online infertility community)	– Semi‐structured interviews	– Online community has advantages (support from peers, informative)	– Generalizability (one Dutch fertility clinic, could be enthusiastic participants on online community) – Need to be confirmed in future studies with larger samples and within more clinics	8/10 (80.0%) Low risk
16	Langarizadeh, Iran (2022)	Cross‐sectional	– Women – Diagnosed with Infertility	220	34.7	(Mobile‐based nutrition educational application)	– Questionnaire of user interface satisfaction	– Good usability and satisfaction	– Generalizability (Persian, culture, area) – Lack of information needs assessment (future educational content) – Lack of cooperation by some specialists (completing the questionnaire) – Need for development of a nutrition education software	5/8 (62.5%) Moderate risk

Abbreviations: ART, assisted reproduction techniques; BMI, body mass index; CBT, cognitive behavioral therapy; CG, control group; ICSI, intracytoplasmic sperm injection; IG, intervention group; IUI, intrauterine insemination; IVF, in vitro fertilization; NG, natural conception group; OI, ovulation induction; RCT, randomized controlled trial; SC‐DC, self‐compassion training using digital stories group; SCV, self‐compassion training using video group.

^a^ Risk of bias; the number of “yes” responses divided by the total number of items. #1 refers to the risk of bias assessment conducted for the quasi‐experimental design study, while #2 pertains to the risk of bias evaluation for the qualitative design study.

^b^ IG′ and CG′, intervention group and control group without baseline assessments.

### 4.2 Descriptions of Digital Health Interventions for Individuals With Infertility

Among the 16 studies, 43.7% used web‐based interventions (*n* = 7), application‐based programs (*n* = 4), online meetings (*n* = 2), email and text messages (*n* = 2), and online community services (*n* = 1). These interventions ranged from simply posting information on web pages (Jones et al. 2020; Vause et al. 2018) or sending educational content via email and text messages (Hojeij et al. 2023; Oostingh et al. 2020) or via applications (Boedt et al. 2023; Langarizadeh et al. 2022; Timmers et al. 2021) to more interactive approaches such as taking quizzes online (Madeira et al. 2018), completing assignments (Cousineau et al. 2008), forming online interactions with healthcare providers for frequently asked questions (Sparidaens et al. 2023), and participating in online meetings (Cercato et al. 2022; Grunberg et al. 2020; Njogu et al. 2023; van Dongen et al. 2016). Modes of interventions included providing educational content on ART treatments, medication, and side effects (Cousineau et al. 2008; Jones et al. 2020; Madeira et al. 2018; Sparidaens et al. 2023; Timmers et al. 2021; Vause et al. 2018) and various other methods, such as cognitive behavioral therapy programs (Sexton et al. 2010; van Dongen et al. 2016), dramatherapy (Cercato et al. 2022), self‐compassion‐based programs (Njogu et al. 2023), mindfulness (Boedt et al. 2023), and coaching on nutrition and health behaviors (Hojeij et al. 2023; Langarizadeh et al. 2022; Oostingh et al. 2020) (Table 1). The time per session varied by intervention, from 5 min to unlimited access, due to differences in accessibility; the duration of the interventions ranged from 2 to 24 weeks. Some were completed in a single intervention (*n* = 2), whereas others involved multiple interventions over a set period (*n* = 7) or were offered unlimited access (*n* = 6). Of the 14 interventions, 9 interventions (64.2%) provided tailored information or feedback based on initial or continuous patient screening regarding the patient's needs and health conditions (Table 2).

**TABLE 2:** Intervention description and replication (TIDieR).^†^

No.	Author (year)	Name of intervention	Why	What	Who provided	How	When and how much	Tailoring
1	Vause et al. (2018)	Web IVF education session	To increase knowledge and reduce stress	– Educational contents IVF protocol, medications, risks of IVF	Not described	Web‐based teaching tool	Unlimited access with 30–40 min required to complete	N/A
2	Grunberg et al. (2020)	Online infertility peer supporter training program	To provide online peer support training for infertility peer supporters	– Peer support training manual contents role of peer supporter, information about infertility, guidelines, monitoring method, frequently asked questions, medical terms, and abbreviations – Training webinar (35 min) – Responding practice	Clinical psychologist, family therapist, clinical social worker	Online meeting platform (GoToMeeting) and YouTube email	4 h	N/A
3	Cercato et al. (2022)	Digital integrated dramatherapy	To help women face very difficult emotions by promoting creativity and internal resources	– Synchronous meeting group building in the digital setting introduce self with a self‐portrait – Psychotherapist meetings (3 times) conception, creation, and dramatization of a personalized mask – Multimedia (13–15 themes) – Conclusive focus group meeting elaborating the individual path in the context and with the interaction of the group	Psychologists	PSYDIT (web‐based psychological support platform)	3 months	Personalized e‐therapy program
4	van Dongen et al. (2016)	Digicoach	To reduce anxiety and depression after first unsuccessful ART cycle	– Psychoeducation and CBT program – Module contents stress management, depressed mood, social support – Online meeting with e‐therapist	Counselor	Web‐based program Online meeting platform	Weekly access to one session and contact with an e‐therapist throughout the entire ART cycle	Personalized e‐therapy program
5	Hojeij et al. (2023)	Smarter pregnancy coaching program	To improve nutritional and lifestyle behaviors of women attempting pregnancy	– Personalized coaching on nutrition and lifestyle behavior – Coaching contents feedback, recommendations, tips, vouchers, seasonal recipes	Not described	email	Weekly emails for 24 weeks	tailored coaching
6	Njogu et al. (2023)	Self‐compassion‐based program	To reduce anxiety and depression symptoms from fertility treatment	– Video contents (SCV) meditation, animated psychoeducation – Online sessions (SC‐DS)	Nurse	Video (SCV) Online meeting platform (Zoom) (SC‐DS)	For 8 weeks, SCV includes two daily 5‐min videos at their convenience, while SC‐DS includes 1‐h sessions	N/A
7	Jones et al. (2020)	Online fertility educational material	To provide educational material for infertility patients	– Educational materials (video, text‐based) causes and treatment of infertility, polycystic ovarian syndrome, fertility medications, ARTs, and optimizing natural fertility.	Not described	Clinic website Facebook and Twitter sites	Unlimited access	N/A
8	Oostingh et al. (2020)	Smarter Pregnancy coaching program	To improve nutritional and lifestyle behaviors undergoing IVF/ICSI	– Personalized coaching on nutrition and lifestyle behavior – Coaching contents tips, recommendations, vouchers, seasonal recipes, feedback on progress	Not described	Email and text messages	Three emails or text messages per week for 24 weeks	Personalized coaching
9	Timmers et al. (2021)	Patient Journey App	To educate patients on fertility medication	– Provided timely information to patients – Contents administer hormone injections, manage side effects	Not described	Smartphone application	Unlimited access	Personalized timing
10	Madeira et al. (2018)	Engaged MD program	To educate patients treatments to prepare for consent documentation	– Multi‐media platform with e‐learning system (video, brief matching quiz) – Module contents IVF, OI‐IUI treatments	Medical providers	Multi‐media platform (video, quiz)	Each video takes 5–7 min Total IVF module: 1 h 20 min Total OI‐IUI module: 50 min	Customize educational contents with e‐sign of informed consent documents
11	Sexton et al. (2010)	WCWI Web version of CWI (coping with infertility)	To reduce general and infertility‐specific stress who are receiving infertility treatments	– CBT based contents and interventions psychoeducation, behavioral skills, cognitive restructuring, other coping skills, personalized coping plan	Not described	Web‐based intervention	For 2 weeks	Personalized coping plan and feedback
12	Boedt et al. (2023)	PreLiFe program	To improve healthy lifestyle and IVF success rates in couples undergoing IVF	– Tailored advice and skills training on diet, physical activity, mindfulness, medical treatment, and medication instructions	Healthcare professional	Mobile application, text messages, and telephone	For 6 months	Tailored advice via text messages and telephone interaction
13	Sparidaens et al. (2023)	myFertiCare	To improve knowledge about infertility and treatment, experiences related to burden of infertility and patient‐centered care	– Provides personalized information and interactive functionalities with doctors and fellow patients	Doctors	Online application	Unlimited access	Tailored counseling
14	Cousineau et al. (2008)	Infertility source	To reduce infertility distress, decisional conflict, marital cohesion and improve infertility self‐efficacy and coping style	– Provide information and stress management techniques, let participants complete a “Confidence Check,” and provide targeted feedback based on the confidence level	Health providers	Web‐based program	Unlimited access	Tailored program and contents
16	Langarizadeh et al. (2022)	Mobile‐based nutrition educational application	To provide nutrition education for improving the quality of life of women with infertility	– Educational contents definitions, disease and treatment instructions, diet and nutrition, dietary habits, personal activities and habits, menstruation status	Not described	Mobile application	Unlimited access	N/A

Abbreviations: ART, assisted reproduction techniques; CBT, cognitive behavioral therapy; ICSI, intracytoplasmic sperm injection; IUI, intrauterine insemination; IVF, in vitro fertilization; OI, ovulation induction; SC‐DC, self‐compassion training using digital stories group; SCV, self‐compassion training using video group.

^†^ Analysis excluding qualitative research; No. 15. Aarts et al. (2015).

### 4.3 Outcomes of Digital Health Interventions

**FIGURE 2:**
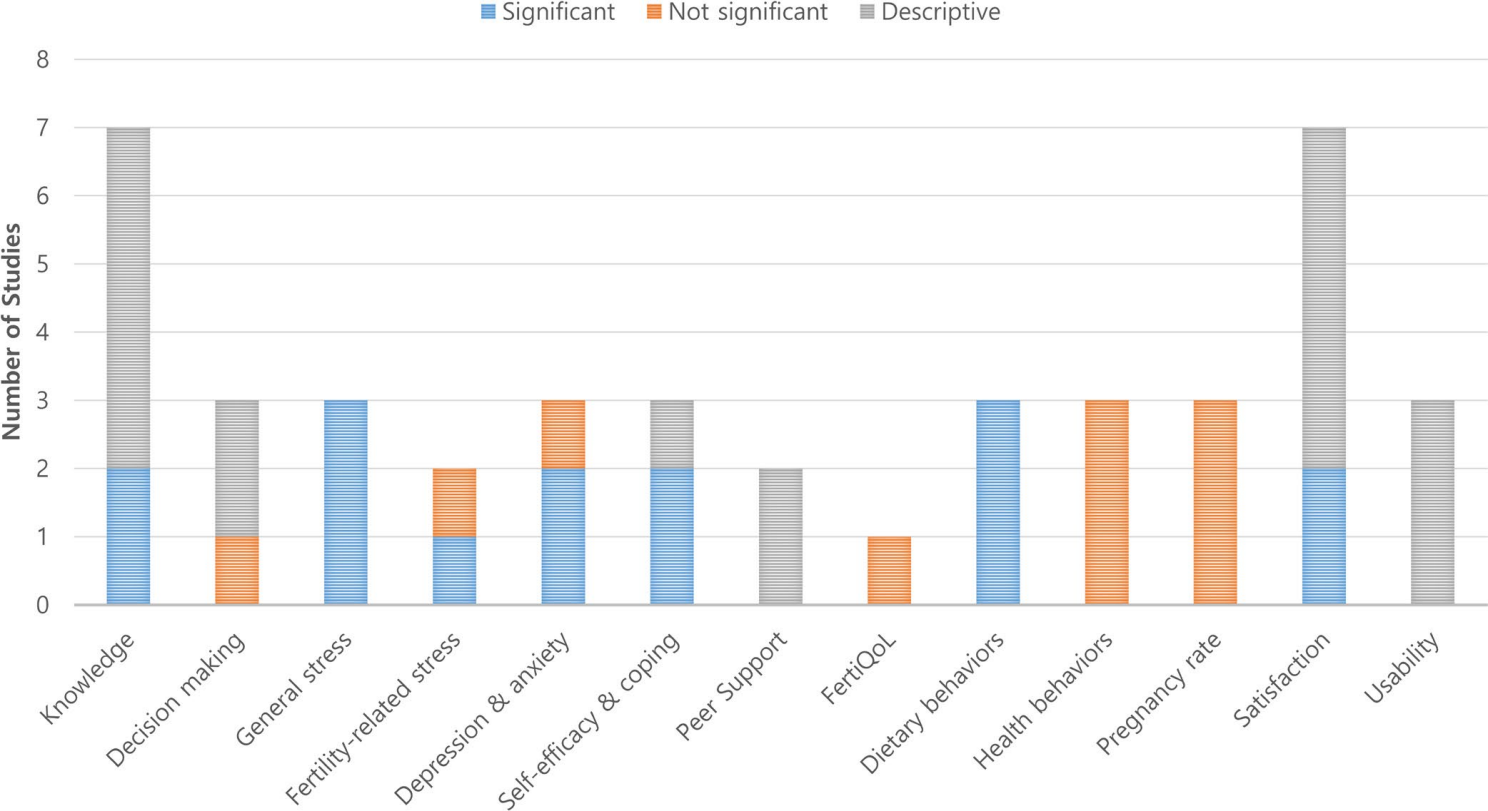
Effectiveness of complementary interventions using technology for infertility. ^†^The colors in the graph differentiate between studies with significant, nonsignificant, and descriptive results. FertiQOL, fertility quality of life.

All studies reported different outcomes when evaluating the effectiveness of interventions in individuals with infertility. We categorized them into 13 distinguishable outcomes. Aligning with the intervention categories, informational outcomes included knowledge and decision‐making; psychological outcomes included general stress, fertility‐related stress, depression and anxiety, self‐efficacy and coping, peer support, and fertility quality of life (FertiQoL); and physical outcomes included dietary behaviors, health behaviors (physical activity, smoking, and alcohol consumption), and pregnancy rate. Satisfaction with the program and its usability was also reported as outcomes (Figure 2 and Table 2).

#### 4.3.1 Informational Outcomes

Informational outcomes were reported in 7 (43.7%) of the 16 studies; these reported that the interventions positively impacted participants' knowledge. However, only two of these studies reported statistically significant results (Timmers et al. 2021; Vause et al. 2018), while the others reported descriptive outcomes. One study reported that, as participants' understanding improved, the time required for healthcare professionals to provide explanations decreased (Madeira et al. 2018). Vause et al. (2018) observed increased knowledge following digital interventions, with no significant difference compared to face‐to‐face education, suggesting that digital interventions are equally effective. In addition, when timely information is provided according to a patient's IVF schedule, knowledge scores increase significantly (Timmers et al. 2021). Two studies indicated that the intervention facilitated better post‐intervention medical decision‐making (Jones et al. 2020; Madeira et al. 2018). However, a study that statistically tested the results found no significant effects (Cousineau et al. 2008), highlighting the need for further validation.

#### 4.3.2 Psychological Outcomes

In 9 of the 16 studies (56.2%), psychological outcomes were reported in individuals with infertility. The interventions were effective for general stress (Cousineau et al. 2008; Sexton et al. 2010; Vause et al. 2018) but showed mixed results for fertility‐related stress; Cousineau et al. (2008) reported a decrease, while Sexton et al. (2010) found no effect. Depression and anxiety decreased in two studies (Njogu et al. 2023; van Dongen et al. 2016) that included psychoeducation. However, one study that focused only on mindfulness (Boedt et al. 2023) found no significant differences. Self‐efficacy and coping significantly improved in two studies that offered interventions such as psychoeducation (Njogu et al. 2023) and stress management (Cousineau et al. 2008). In addition, studies that provided online doctor–patient interactions (Sparidaens et al. 2023) reported positive descriptive outcomes. Regarding peer support, studies reported that individuals with infertility felt supported by their peers in an online community (Aarts et al. 2015), and online group therapy (Cercato et al. 2022) showed that peer support could also be fostered through online interactions. Finally, only Boedt et al. (2023) examined the effect of digital interventions on fertility‐related quality of life; however, the effect was not significant.

#### 4.3.3 Physical Outcomes

Physical outcomes were reported in 4 of the 16 studies (25.0%). Three studies implemented CITs that focused on dietary behaviors, including fruit and vegetable intake (Boedt et al. 2023; Hojeij et al. 2023; Oostingh et al. 2020). These studies also addressed health behaviors such as smoking, alcohol consumption, and physical activity. Although positive changes in dietary behaviors have been reported, no significant effects on health behaviors have been found. In addition, three studies investigated whether CITs could improve pregnancy rates (Boedt et al. 2023; Njogu et al. 2023; Oostingh et al. 2020), but no significant results were found. Interventions aimed at improving nutritional and lifestyle behaviors (Oostingh et al. 2020), reducing anxiety and depression symptoms (Njogu et al. 2023), and integrating knowledge about treatment, mindfulness, and a healthy lifestyle (Boedt et al. 2023) did not significantly increase pregnancy rates.

#### 4.3.4 Satisfaction and Usability

Of the 16 studies, 8 reported on participant satisfaction and intervention usability. In seven studies, participants expressed high satisfaction with the program (Aarts et al. 2015; Cousineau et al. 2008; Grunberg et al. 2020; Jones et al. 2020; Madeira et al. 2018; Timmers et al. 2021; Vause et al. 2018). However, only two studies (Timmers et al. 2021; Vause et al. 2018) provided statistically verified results, while the others presented only descriptive findings. Regarding usability, three studies reported descriptive results, indicating the high usability of the digital media used (Cercato et al. 2022; Langarizadeh et al. 2022; Sparidaens et al. 2023). Overall, the studies reported high satisfaction and usability regardless of the content or technology used. No studies have addressed these specific issues or barriers.

## 5 Discussion

In this study, we investigated the types of digital health complementary interventions developed for individuals with infertility; their effectiveness on informational, psychological, and physical outcomes; as well as their limitations.

Among the 16 studies, we found that only 7 provided interventions for both men and women; among them, only 4 were conducted for couples with infertility. Kianfar et al. (2024) reported that the positive performance of husbands with financial and emotional support and reduced marital conflict was important for women with infertility to cope with the situation. Furthermore, counseling for couples with infertility is more effective than counseling for individuals with infertility in relieving infertility distress (Koser 2020). These studies suggest that complementary interventions are necessary for couples with infertility to improve health‐related outcomes. In addition, most of the research was conducted in Europe and the United States. Although the infertile population has been growing continuously in the Asia‐Pacific region (Luo et al. 2024), little digital health research has been conducted in the region on infertility, suggesting a lack of CITs tailored to Asian populations. As cultural perceptions of infertility and the need for cultural sensitivity (e.g., societal pressure and family inheritance) are diverse across different ethnicities, it is imperative to develop customized interventions for each (Kuug et al. 2023). Regarding study design, only 8 of the 16 studies were RCTs, and validation of health‐related outcomes for CITs was insufficient. Furthermore, some studies did not report the results for all measured variables; most studies only verified the effects after the intervention rather than comparing the effectiveness before and after the intervention. In addition, only nine studies were considered to have low risk of bias (70% of the “yes” responses in the critical appraisal checklist) (Goplen et al. 2019). Thus, high‐quality experimental studies (e.g., RCTs or quasi‐experimental studies) are required to evaluate the effectiveness of CITs in individuals with infertility.

Providing information about infertility treatments, medications, and side effects has been shown to improve treatment understanding and help in decision‐making regarding ARTs (Timmers et al. 2021). As delays in childbearing can lead to regret and depression among individuals with infertility, healthcare professionals should raise infertility awareness and provide information on ARTs to support decision‐making, thereby preventing such delays (Adachi et al. 2020). In addition, understanding patient priorities regarding the effectiveness, safety, burden, and cost of ARTs is crucial for offering patient‐centered approaches and reducing the stress associated with infertility treatment. In our review, seven studies reported fertility‐related knowledge and decision‐making as intervention outcomes. Timmers et al. (2021) revealed that providing timely information to patients via apps was useful during infertility treatment to inform them about the related procedures and medications. However, the included studies used non‐validated and disparate questionnaires, leading to inconsistencies in measuring the knowledge deficiency regarding the infertility treatments, before the informational intervention and its after‐effects. Furthermore, not only knowledge but also attitudes toward ARTs have a favorable impact on the practice and persistence of technology (Demissei et al. 2024). Thus, we propose the development of tools to evaluate knowledge, attitudes, and practices based on the informational needs of couples with infertility regarding ARTs.

Psychological aspects were prevalent in 25%–40% of individuals with infertility, and higher anxiety and depression were reported in them, compared to fertile controls (Rooney and Domar 2018). Furthermore, a negative association between mental disorders and fertility rate was reported in a previous study (Szkodziak et al. 2020). These studies highlight that psychometric factors should be measured as outcomes to effectively manage the psychological health of couples with infertility. In our review, we found nine studies reporting psychological factors as outcomes of the interventions. Self‐compassion, coping skills, and psychoeducation programs delivered via web or app have been shown to improve self‐efficacy and coping skills in individuals with infertility, while also reducing stress and anxiety. However, only 1 of the 16 studies reported on FertiQoL and reported no such effect. In addition, only two studies focused on fertility‐related stress, and the results were inconsistent. FertiQoL is a well‐designed tool for individuals experiencing fertility challenges or treatments, which consists of personal (emotional, social, relational, and mind/body) and treatment (tolerability and environment) domains. In a previous study, longer infertility duration and less patient‐centered care negatively impacted FertiQoL, whereas psychological interventions had a positive impact (Koert et al. 2021). Boedt et al. (2023), who applied digital interventions, including mindfulness and in‐person mindfulness‐based physical or psychological programs lasting for 2–3 months during IVF, showed improvements in FertiQoL, reduced stress levels, and enhanced treatment tolerability (Huerta‐De La Luz et al. 2024). Given these gaps, further research should evaluate the effectiveness of digital interventions on FertiQoL or fertility‐related stress and demonstrate the impact of such interventions on infertility‐related psychological outcomes. Although online communication offers peer support, its effectiveness has yet to be established. Although online communities allow individuals with infertility to share their experiences and support each other, feelings of isolation can persist. Therefore, involving peer volunteers and professional coordination is necessary to enhance community effectiveness.

Lifestyle changes, such as addressing weight, diet, alcohol use, tobacco use, physical activity, stress, and sleep, can help couples stay engaged in treatment; enhance their physical and mental health; and potentially increase pregnancy success rates (Dupont et al. 2020). Previous research has indicated that obesity could negatively affect ovulation induction and reduce clinical pregnancy and live birth rates after ART interventions, and that body mass index is a significant factor affecting pregnancy outcomes (McKinnon et al. 2016). These findings suggest that lifestyle changes accompanying successful physiological changes (e.g., weight reduction) are critical for enhancing pregnancy rates. In our review, four studies focused on physical support; however, they showed no significant improvements in health behaviors such as smoking, drinking, physical activity, lifestyle changes, or pregnancy rates. Therefore, future studies should investigate the optimal timing and duration of lifestyle interventions before ART interventions and assess the cumulative pregnancy rates across multiple cycles. Furthermore, we propose the development of objective biomarkers for predicting embryo and uterine reproductive competence and physical and psychological states (e.g., cortisol, α‐amylase, and proinflammatory cytokines) to better evaluate the effectiveness of these interventions.

Notably, dropout rates can be as high as 70%, primarily owing to the physical and emotional burdens of the treatment (Ghorbani et al. 2020). Enhancing fertility awareness and treatment‐related literacy through online materials can positively impact knowledge and perceived susceptibility, further supporting successful outcomes (Herzberger et al. 2022). Furthermore, complementary interventions can reduce treatment dropout and improve the quality of life (Dupont et al. 2020). Therefore, it is crucial to continue providing informational, physical, and psychological support through digital interventions to reduce ART discontinuation rates and improve pregnancy rates.

Digital health interventions use ICT to provide tailored, cost‐effective, and sustainable care to meet individual health‐related needs (Kowatsch and Fleisch 2021). Incorporating eHealth, wearable devices, and couple‐based approaches may further support individuals throughout infertility treatment (Hoek et al. 2022). In our study, the modes of digital health primarily utilized web‐based platforms, applications, online communities, and email; however, email‐based interventions that provided one‐sided information were not significantly effective. One‐way information delivery via email tends to be less effective in promoting behavioral change or improving psychological outcomes because it lacks the important two‐way interactivity for supporting individuals with infertility. Methods such as virtual support groups or telehealth consultations may be more effective as interactive alternatives to email‐based interventions.

The duration of CITs for infertility varied; for example, knowledge‐based interventions were delivered for 30 min to 4 h, whereas physical–psychological interventions were delivered for 2–6 months or throughout the ART cycle. However, in our review, longer interventions lasting 6 months did not successfully improve health outcomes owing to higher dropout rates (Boedt et al. 2023). This is similar to the previous studies reporting that longer interventions, such as those lasting 6 months before IVF, did not improve embryo utilization or cumulative live birth rates (Wang et al. 2021). In contrast, a 3‐month face‐to‐face intervention, including diet, exercise, and psychological counseling, improved health behaviors, reduced treatment cycles, and potentially increased pregnancy rates (Sim et al. 2014). Therefore, additional intervention studies of various durations are required to determine the optimal duration of digital interventions to balance adherence to the intervention and its effectiveness.

In this review, complex interventions were delivered primarily by clinical psychologists, social workers, or healthcare providers without a multidisciplinary approach. This underscores the need for future research to include interdisciplinary teams comprising physicians, nurses, psychologists, and exercise specialists to provide comprehensive and holistic support. Although CITs show promise for improving informational, psychological, and physical outcomes in individuals with infertility, several barriers hinder their effective implementation. These include limited interactivity in one‐way communication methods such as email, a lack of standardized tools for outcome assessment, and limited involvement of multidisciplinary teams. Moreover, despite evidence suggesting that couple‐based interventions may enhance coping with infertility treatments, CITs specifically designed for couples are lacking (Sparidaens et al. 2023). Furthermore, the Asia‐Pacific region, with rising infertility rates, has experienced limited development and application of CITs. This highlights the need for comprehensive CITs that are applicable to diverse populations. It is also essential to develop technology‐based complementary interventions to address the specific needs of couples with infertility, support interactive communication, and incorporate a multidisciplinary approach. The effectiveness of such interventions should be evaluated using validated tools, with the goal of improving fertility‐related quality of life, reducing infertility‐related stress, and ultimately enhancing pregnancy outcomes.

## 6 Limitations

This review included all eligible studies, regardless of the heterogeneity of their design, including RCTs and quasi‐experimental, cross‐sectional, and qualitative studies. Studies with a high risk of bias were also included. Therefore, caution should be exercised when interpreting and applying these results. Furthermore, some studies failed to report specific outcomes, and the lack of studies comparing control groups or pre‐ and post‐intervention effects further limited our conclusions regarding the effectiveness of digital interventions for individuals with infertility. In addition, many studies had small sample sizes and focused on specific geographical regions, making it difficult to generalize the results to other populations. Given these limitations, future research should prioritize RCTs that include comprehensive digital interventions to address the physical, psychological, and informational needs of couples with infertility.

## 7 Conclusion

This review demonstrates that various CITs have been developed for individuals with infertility, and studies have measured their effectiveness on informational, psychological, and physical outcomes. Favorable improvements in knowledge, general stress, depression and anxiety, self‐efficacy and coping, dietary behaviors, and satisfaction were found; however, FertiQoL, health behavior, and pregnancy rates were not significantly affected. Given that the digital method of intervention, time and duration of intervention, and providers varied among the studies, further reviews are needed to determine effective ways of managing the needs of couples with infertility undergoing or planning to undergo ART interventions. In addition, large‐scale RCTs are required to evaluate the effectiveness of CITs. A multidisciplinary approach involving nurses should be employed when providing CITs to couples with infertility to enhance health outcomes and reduce dropout rates of ART interventions.

## 8 Relevance for Clinical Practice

Several complementary interventions incorporating various technologies were developed, and they demonstrated statistically significant improvements in outcomes such as knowledge, stress, depression, and anxiety, self‐efficacy, coping, and dietary behaviors. Therefore, we propose implementing these interventions for individuals undergoing ART to help reduce their stress and anxiety levels while supporting the development of effective coping strategies. Furthermore, these interventions can be effectively utilized in clinical settings to deliver educational resources, potentially alleviating the burden of repetitive explanations typically required of healthcare providers. However, it is important to note the current lack of fertility‐specific assessment tools and the need to develop interventions that adopt individualized and multidisciplinary approaches, particularly those aimed at achieving long‐term outcomes such as sustained health behavior changes and improved pregnancy rates. To establish effective strategies to manage the burden of infertility, we recommend the application of these interventions and their evaluation through large‐scale RCTs.

## Author Contributions

**Jiwon Lee:** conceptualization (lead), methodology (supporting) – data extraction and analysis, writing – original draft, review and editing. **Jaejin Kang:** Conceptualization (supporting), methodology (supporting) – data extraction and analysis, visualization, writing – original draft, review and editing. **Jo Woon Seok:** conceptualization (supporting), methodology (lead) – data extraction and analysis, writing – original draft, review and editing.

## Ethics Statement

The authors have nothing to report.

## Conflicts of Interest

The authors declare no conflicts of interest.

## Supporting information


**Data S1:** Supporting Information.

**Table S1:** Search strategies in databases.

## Data Availability

Data sharing is not applicable to this article as no new data were created or analyzed in this study.
