# Supplementary material for: Complementary Interventions Using Technology for Individuals With Infertility Using Assisted Reproductive Technology: A Scoping Review
Source: Nurs Health Sci. 2025 Sep 5;27(3):e70227. doi: 10.1111/nhs.70227 (PMC12413509; doi:10.1111/nhs.70227)
Supplement: Supplementary file 2 — Table S1: Search strategies in databases. [file NHS-27-e70227-s002.docx]

**Appendix A**

**Supplementary Table. Search Strategies in Databases**

1. **PubMed Search Trail**

| Search # | MeSH Terms and Key Words | Articles No. |
| --- | --- | --- |
| #1 | (Reproductive Technic?[TW] OR Assisted Reproductive Technique?[TW] OR Assisted Reproductive Technic?[TW] OR Assisted Reproductive Technolog*[TW] OR Reproductive Technolog*[TW] OR Embryo Transfer[TW] OR Fertility Preservation[TW] OR Fertilization in Vitro[TW] OR Insemination, Artificial[TW] OR Ovulation Induction[TW] OR Superovulation[TW] OR Reproductive procedure[TW] OR Infertility therapy[TW] OR Reproductive Techniques[MH] OR Reproductive Techniques, Assisted[MH]) | 180,163 |
| #2 | (Complementary therap*[TW] OR Complementary Medicine[TW] OR Alternative Medicine[TW] OR Acupuncture Therapy[TW] OR Acupotom*[TW] OR Anthroposophy[TW] OR Holistic Health[TW] OR ?Holistic Therap*[TW] OR ?Holistic Health[TW] OR Mind?Body Therap*[TW] OR Aromatherapy[TW] OR Psychotherap*[TW] OR Meditation[TW] OR Mental Healing[TW] OR Relaxation Therap*[TW] OR Therapeutic Touch[TW] OR Yoga[TW] OR Aromatherapy[TW] OR Sensory Art Therap*[TW] OR Art Therap*[TW] OR Color Therap*[TW] OR Dance Therap*[TW] OR Music Therap*[TW] OR Spiritual Therap*[TW] OR Counceling[TW] OR E?Counseling[TW] OR E-Therapy[TW] OR Remote Consultation[TW] OR Sex Counseling[TW] OR Cognitive Behavioral Therap*[TW] OR Cognitive Therap*[TW] OR Psychotherap*[TW] OR diet therapy[TW] OR kinesiotherapy[TW] OR Kinesitherapy [TW] OR kinesiatrics[TW] OR sport[TW] OR Behavior therap*[TW] OR Rehabilitation[TW] OR Habilitation[TW] OR Education[TW] OR Group?, Self-Help[TW] OR Self?Help Group?[TW] OR Support Group?[TW] OR Group?, Support[TW] OR Club?, Therapeutic Social[TW] OR Social Club?, Therapeutic[TW] OR Therapeutic Social Club?[TW] OR Behavior Therapies[TW] OR Behavior Treatment[TW] OR Treatment, Behavior[TW] OR Therapy, Conditioning[TW] OR Conditioning Therap*[TW] OR Therapy, Behavior[TW] OR Behavior Change Techniq*[TW] OR Technique, Behavior Change[TW] OR Behavior Modification?[TW] OR Modification, Behavior[TW] OR Complementary Therapies[MH] OR Acupuncture Therapy[MH] OR Holistic Health[MH] OR Yoga[MH] OR Aromatherapy[MH] OR Counseling[MH] OR Distance Counseling[MH] OR Remote Consultation[MH] OR Cognitive Behavioral Therapy[MH] OR Cognitive therapy[MH] OR Psychotherapy, group[MH] OR Nutrition Therapy[MH] OR Behavior therapy[MH] OR Behavioral Disciplines and Activities[MH] OR Rehabilitation[MH] OR Education[MH] OR Self-help groups[MH] OR Behavior therapy[MH]) | 3,096,126 |
| #3 | (Digital health[TW] OR Digital technolog*[TW] OR Health technolog*[TW] OR Internet Based Intervention*[TW] OR Internet-based[TW] OR Internet intervention*[TW] OR Web-based[TW] OR Web?based intervention*[TW] OR Computer-based[TW] OR Computer Assisted[TW] OR Computer based[TW] OR Computer mediated[TW] OR Online intervention*[TW] OR Online[TW] OR Mobile App*[TW] OR Portable Software App*[TW] OR Software App*[TW] OR Smartphone App*[TW] OR Application*[TW] OR Portable Electronic App*[TW] OR Electronic App*[TW] OR Mobile Health[TW] OR Telemedicine[TW] OR Tele-medicine[TW] OR mHealth[TW] OR m-health[TW] OR Remote Consultation[TW] OR Telenursing[TW] OR Telehealth[TW] OR eHealth[TW] OR e-health[TW] OR Telecare[TW] OR Telehealth[TW] OR Assistive technolog*[TW] OR Smart[TW] OR Digital[TW] OR Virtual[TW] OR Media[TW] OR Touchscreen[TW] OR iPad[TW] OR Mobile[TW] OR Tablet*[TW] OR E?Therap*[TW] OR Information Technology[TW] OR ICT[TW] OR Information communication technolog*[TW] OR Health Communication[TW] OR Wireless technolog*[TW] OR Wearable*[TW] OR Telecommunication*[TW] OR Information technology device[TW] OR Communication software[TW] OR Biomedical software[TW] OR Healthcare software[TW] OR Self-care software[TW] OR Interactive voice response system[TW] OR Digital technology[MH] OR Internet-Based Intervention[MH] OR Internet[MH] OR Mobile Applications[MH] OR Telemedicine[MH] OR Multimedia[MH] OR Telenursing[MH] OR Information Technology[MH] OR Wireless Technology[MH]) | 3,200,372 |
| #4 | #1 AND #2 AND #3 | 1,266 |
| #5 | #5 AND English[lang] NOT (animals[Mesh:noexp] NOT (animals[Mesh:noexp] AND humans[Mesh])) NOT (Autobiography[ptyp] OR Bibliography[ptyp] OR Biography[ptyp] OR pubmed books[filter] OR Comment[ptyp] OR Dataset[ptyp] OR Dictionary[ptyp] OR Editorial[ptyp] OR Electronic Supplementary Materials[ptyp] OR Interview[ptyp] OR Legislation[ptyp] OR News[ptyp] OR Newspaper Article[ptyp] OR Retracted Publication[sb] OR Retraction of Publication[sb] OR Technical Report[ptyp] OR Letter[ptyp] OR Review[ptyp]) AND (2000/01/01[PDAT] : 2023/12/31[PDAT]) | 696 |

1. **CINHAL Search Trail**

| Search # | Sub Headings (MH) and Key Words | Articles No. |
| --- | --- | --- |
| #1 | ((Reproductive Technic? OR Assisted Reproductive Technique? OR Assisted Reproductive Technic? OR Assisted Reproductive Technolog* OR Reproductive Technolog* OR Embryo Transfer OR Fertility Preservation OR Fertilization in Vitro OR Insemination, Artificial OR Ovulation Induction OR Superovulation OR Reproductive procedure OR Infertility therapy) OR MH(Reproductive Techniques OR Reproductive Techniques, Assisted)) | 21,468 |
| #2 | ((Complementary therap* OR Complementary Medicine OR Alternative Medicine OR Acupuncture Therapy OR Acupotom* OR Anthroposophy OR Holistic Health OR ?Holistic Therap* OR ?Holistic Health OR Mind?Body Therap* OR Aromatherapy OR Psychotherap* OR Meditation OR Mental Healing OR Relaxation Therap* OR Therapeutic Touch OR Yoga OR Aromatherapy OR Sensory Art Therap* OR Art Therap* OR Sensory Art Therap* OR Color Therap* OR Dance Therap* OR Music Therap* OR Spiritual Therap* OR Counceling OR E?Counseling OR E-Therapy OR Remote Consultation OR Sex Counseling OR Cognitive Behavioral Therap* OR Cognitive Therap* OR Psychotherap* OR diet therapy OR kinesiotherapy OR Kinesitherapy OR kinesiatrics OR sport OR Behavior therap* OR Rehabilitation OR Habilitation OR Education OR Group?, Self-Help OR Self?Help Group? OR Support Group? OR Group?, Support OR Club?, Therapeutic Social OR Social Club?, Therapeutic OR Therapeutic Social Club? OR Behavior Therapies OR Behavior Treatment OR Treatment, Behavior OR Therapy, Conditioning OR Conditioning Therap* OR Therapy, Behavior OR Behavior Change Techniq* OR Technique, Behavior Change OR Behavior Modification? OR Modification, Behavior) OR MH(Complementary Therapies OR Acupuncture Therapy OR Holistic Health OR Yoga OR Aromatherapy OR Counseling OR Distance Counseling OR Remote Consultation OR Cognitive Behavioral Therapy OR Cognitive therapy OR Psychotherapy, group OR Nutrition Therapy OR Behavior therapy OR Behavioral Disciplines and Activities OR Rehabilitation OR Education OR Self-help groups OR Behavior therapy)) | 1,226,630 |
| #3 | ((Digital health OR Digital technolog* OR Health technolog* OR Internet Based Intervention* OR Internet-based OR Internet intervention* OR Web-based OR Web?based intervention* OR Computer-based OR Computer Assisted OR Computer based OR Computer mediated OR Online intervention* OR Online OR Mobile App* OR Portable Software App* OR Software App* OR Smartphone App* OR Application* OR Portable Electronic App* OR Application* OR Electronic App* OR Mobile Health OR Telemedicine OR Tele-medicine OR mHealth OR m-health OR Remote Consultation OR Telenursing OR Telehealth OR eHealth OR e-health OR Telecare OR Telehealth OR Assistive technolog* OR Smart OR Digital OR Virtual OR Media OR Touchscreen OR iPad OR Mobile OR Tablet* OR E?Therap* OR Information Technology OR ICT OR Information communication technolog* OR Health Communication OR Wireless technolog* OR Wearable* OR Telecommunication* OR Information technology device OR Communication software OR Biomedical software OR Healthcare software OR Self-care software OR Interactive voice response system) OR MH(Digital technology OR Internet-Based Intervention OR Internet OR Mobile Applications OR Telemedicine OR Multimedia OR Telenursing OR Information Technology OR Wireless Technology)) | 684,618 |
| #4 | #1 AND #2 AND #3 | 227 |
| #5 | #4 Limiters: 2000-2023, (English), academic journal | 207 |

1. **Embase Search Trail**

| Search # | Emtree Term and Key Words | Articles No. |
| --- | --- | --- |
| #1 | ‘Reproductive Technic?’:ab,ti,tn OR ‘Assisted Reproductive Technique?’:ab,ti,tn OR ‘Assisted Reproductive Technic?’:ab,ti,tn OR ‘Assisted Reproductive Technolog*’:ab,ti,tn OR ‘Reproductive Technolog*’:ab,ti,tn OR ‘Embryo Transfer’ OR ‘Fertility Preservation’:ab,ti,tn OR ‘Fertilization in Vitro’:ab,ti,tn OR ‘Insemination, Artificial’ OR ‘Ovulation Induction’:ab,ti,tn OR Superovulation OR ‘Reproductive procedure’:ab,ti,tn OR ‘Infertility therapy’:ab,ti,tn OR ‘reproductive procedure’/exp OR ‘infertility therapy’/exp | 172,962 |
| #2 | ‘Complementary therap*’:ab,ti,tn OR ‘Complementary Medicine’:ab,ti,tn OR ‘Alternative Medicine’:ab,ti,tn OR ‘Acupuncture Therapy’:ab,ti,tn OR Acupotom*:ab,ti,tn OR Anthroposophy:ab,ti,tn OR ‘Holistic Health’ Acupotom*:ab,ti,tn OR ‘?Holistic Therap*’:ab,ti,tn OR ‘?Holistic Health’:ab,ti,tn OR ‘Mind?Body Therap*’:ab,ti,tn OR Aromatherapy:ab,ti,tn OR Psychotherap*:ab,ti,tn OR Meditation:ab,ti,tn OR ‘Mental Healing’:ab,ti,tn OR ‘Relaxation Therap*’:ab,ti,tn OR ‘Therapeutic Touch’:ab,ti,tn OR Yoga:ab,ti,tn OR Aromatherapy:ab,ti,tn OR ‘Sensory Art Therap*’:ab,ti,tn OR ‘Art Therap*’:ab,ti,tn OR ‘Color Therap*’:ab,ti,tn OR ‘Dance Therap*’:ab,ti,tn OR ‘Music Therap*’:ab,ti,tn OR ‘Spiritual Therap*’:ab,ti,tn OR Counceling:ab,ti,tn OR E?Counseling:ab,ti,tn OR E-Therapy:ab,ti,tn OR ‘Remote Consultation’:ab,ti,tn OR ‘Sex Counseling’:ab,ti,tn OR ‘Cognitive Behavioral Therap*’:ab,ti,tn OR ‘Cognitive Therap*’:ab,ti,tn OR Psychotherap*:ab,ti,tn OR ‘diet therapy’:ab,ti,tn OR kinesiotherapy:ab,ti,tn OR Kinesitherapy:ab,ti,tn OR kinesiatrics:ab,ti,tn OR sport:ab,ti,tn OR ‘Behavior therap*’:ab,ti,tn OR Rehabilitation:ab,ti,tn OR Habilitation:ab,ti,tn OR Education:ab,ti,tn OR ‘Group?:ab,ti,tn, Self-Help’:ab,ti,tn OR ‘Self?Help Group?’:ab,ti,tn OR ‘Support Group?’:ab,ti,tn OR ‘Group?:ab,ti,tn, Support’:ab,ti,tn OR ‘Club?:ab,ti,tn, Therapeutic Social’:ab,ti,tn OR ‘Social Club?:ab,ti,tn, Therapeutic’:ab,ti,tn OR ‘Therapeutic Social Club?’:ab,ti,tn OR ‘Behavior Therapies’:ab,ti,tn OR ‘Behavior Treatment’:ab,ti,tn OR ‘Treatment, Behavior’:ab,ti,tn OR ‘Therapy, Conditioning’:ab,ti,tn OR ‘Conditioning Therap*’:ab,ti,tn OR ‘Therapy, Behavior’:ab,ti,tn OR ‘Behavior Change Techniq*’:ab,ti,tn OR ‘Technique, Behavior Change’:ab,ti,tn OR ‘Behavior Modification?’:ab,ti,tn OR ‘Modification, Behavior’:ab,ti,tn OR ‘alternative medicine’/exp OR ‘psychotherapy’/exp OR ‘counseling’/exp OR ‘cognitive behavioral therapy’/exp OR ‘diet therapy’/exp OR ‘kinesiotherapy’/exp OR ‘sport’/exp OR ‘education’/exp OR | 3,348,907 |
| #3 | ‘Digital health’:ab,ti,tn OR ‘Digital technolog*’:ab,ti,tn OR ‘Health technolog*’:ab,ti,tn OR ‘Internet Based Intervention*’:ab,ti,tn OR Internet-based:ab,ti,tn OR ‘Internet intervention*’:ab,ti,tn OR Web-based:ab,ti,tn OR ‘Web?based intervention*’:ab,ti,tn OR Computer-based:ab,ti,tn OR ‘Computer Assisted’:ab,ti,tn OR ‘Computer based’:ab,ti,tn OR ‘Computer mediated’:ab,ti,tn OR ‘Online intervention*’:ab,ti,tn OR Online:ab,ti,tn OR ‘Mobile App*’:ab,ti,tn OR ‘Portable Software App*’:ab,ti,tn OR ‘Software App*’:ab,ti,tn OR ‘Smartphone App*’:ab,ti,tn OR Application*:ab,ti,tn OR ‘Portable Electronic App*’:ab,ti,tn OR ‘Electronic App*’:ab,ti,tn OR ‘Mobile Health’:ab,ti,tn OR Telemedicine:ab,ti,tn OR Tele-medicine:ab,ti,tn OR mHealth:ab,ti,tn OR m-health:ab,ti,tn OR ‘Remote Consultation’:ab,ti,tn OR Telenursing:ab,ti,tn OR Telehealth:ab,ti,tn OR eHealth:ab,ti,tn OR e-health:ab,ti,tn OR Telecare:ab,ti,tn OR Telehealth:ab,ti,tn OR ‘Assistive technolog*’:ab,ti,tn OR Smart:ab,ti,tn OR Digital:ab,ti,tn OR Virtual:ab,ti,tn OR Media:ab,ti,tn OR Touchscreen:ab,ti,tn OR iPad:ab,ti,tn OR Mobile:ab,ti,tn OR Tablet*:ab,ti,tn OR E?Therap*:ab,ti,tn OR ‘Information Technology’:ab,ti,tn OR ICT:ab,ti,tn OR ‘Information communication technolog*’:ab,ti,tn OR ‘Health Communication’:ab,ti,tn OR ‘Wireless technolog*’:ab,ti,tn OR Wearable*:ab,ti,tn OR Telecommunication*:ab,ti,tn OR ‘Information technology device’:ab,ti,tn OR ‘Communication software’:ab,ti,tn OR ‘Biomedical software’:ab,ti,tn OR ‘Healthcare software’:ab,ti,tn OR ‘Self-care software’:ab,ti,tn OR ‘Interactive voice response system’:ab,ti,tn OR ‘mobile application’/exp OR ‘telehealth’/exp OR ‘mobile phone’/exp OR ‘wearable computer’/exp OR ‘information technology device’/exp OR ‘communication software’/exp OR ‘biomedical software’/exp OR ‘healthcare software’/exp OR ‘self-care software’/exp OR ‘interactive voice response system’/exp OR ‘smart watch’/exp OR ‘activity tracker’/exp OR ‘actigraph’/exp | 3,712,944 |
| #4 | #1 AND #2 AND #3 AND ([article]/lim OR [article in press]/lim OR [conference paper]/lim OR [data papers]/lim OR [short survey]/lim OR [preprint]/lim) AND [humans]/lim AND [english]/lim AND [2000-2023]/py | 513 |

1. **PsychINFO Search Trail**

| Search # | MeSH Terms and Key Words | Articles No. |
| --- | --- | --- |
| #1 | “Reproductive Technic?” OR “Assisted Reproductive Technique?” OR “Assisted Reproductive Technic?” OR “Assisted Reproductive Technolog*” OR “Reproductive Technolog*” OR “Embryo Transfer” OR “Fertility Preservation” OR “Fertilization in Vitro” OR “Insemination, Artificial” OR “Ovulation Induction” OR Superovulation OR “Reproductive procedure” OR “Infertility therapy” OR MAINSUBJECT.EXACT(“Reproductive Techniques”) OR MAINSUBJECT.EXACT(“Reproductive Techniques, Assisted”) | 3,251 |
| #2 | “Complementary therap*” OR “Complementary Medicine” OR “Alternative Medicine” OR “Acupuncture Therapy” OR Acupotom* OR Anthroposophy OR “Holistic Health” OR “Holistic Therap*” OR “Holistic Health” OR “Mind Body Therap*” OR Aromatherapy OR Psychotherap* OR Meditation OR “Mental Healing” OR “Relaxation Therap*” OR “Therapeutic Touch” OR Yoga OR Aromatherapy OR “Sensory Art Therap*” OR “Art Therap*” OR “Color Therap*” OR “Dance Therap*” OR “Music Therap*” OR “Spiritual Therap*” OR Counceling OR “E Counseling” OR E-Therapy OR “Remote Consultation” OR “Sex Counseling” OR “Cognitive Behavioral Therap*” OR “Cognitive Therap*” OR Psychotherap* OR “diet therapy” OR kinesiotherapy OR Kinesitherapy OR kinesiatrics OR sport OR “Behavior therap*” OR Rehabilitation OR Habilitation OR Education OR “Group?, Self-Help” OR “Self?Help Group?” OR “Support Group?” OR “Group, Support” OR “Club, Therapeutic Social” OR “Social Club, Therapeutic” OR “Therapeutic Social Club?” OR “Behavior Therapies” OR “Behavior Treatment” OR “Treatment, Behavior” OR “Therapy, Conditioning” OR “Conditioning Therap*” OR “Therapy, Behavior” OR “Behavior Change Techniq*” OR “Technique, Behavior Change” OR “Behavior Modification?” OR “Modification, Behavior" OR MAINSUBJECT.EXACT(“Complementary Therapies”) OR MAINSUBJECT.EXACT(“Acupuncture Therapy”) OR MAINSUBJECT.EXACT(“Holistic Health”) OR MAINSUBJECT.EXACT(“Yoga”) OR MAINSUBJECT.EXACT(“Aromatherapy”) OR MAINSUBJECT.EXACT(“Counseling”) OR MAINSUBJECT.EXACT(“Distance Counseling”) OR MAINSUBJECT.EXACT(“Remote Consultation”) OR MAINSUBJECT.EXACT(“Cognitive Behavioral Therapy”) OR MAINSUBJECT.EXACT(“Cognitive therapy”) OR MAINSUBJECT.EXACT(“Psychotherapy, group”) OR MAINSUBJECT.EXACT(“Nutrition Therapy”) OR MAINSUBJECT.EXACT(“Behavior therapy”) OR MAINSUBJECT.EXACT(“Behavioral Disciplines and Activities”) OR MAINSUBJECT.EXACT(“Rehabilitation”) OR MAINSUBJECT.EXACT(“Education”) OR MAINSUBJECT.EXACT(“Self-help groups”) OR MAINSUBJECT.EXACT(“Behavior therapy”) | 1454,129 |
| #3 | “Digital health” OR “Digital technolog*” OR “Health technolog*” OR “Internet Based Intervention*” OR “Internet-based” OR “Internet intervention*” OR “Web-based” OR “Web?based intervention*” OR “Computer-based” OR “Computer Assisted” OR “Computer based” OR “Computer mediated” OR “Online intervention*” OR “Online” OR “Mobile App*” OR “Portable Software App*” OR “Software App*” OR “Smartphone App*” OR “Application*” OR “Portable Electronic App*” OR “Electronic App*” OR “Mobile Health” OR “Telemedicine” OR “Tele-medicine” OR “mHealth” OR “m-health” OR “Remote Consultation” OR “Telenursing” OR Telehealth OR eHealth OR e-health OR Telecare OR Telehealth OR “Assistive technolog*” OR Smart OR Digital OR Virtual OR Media OR Touchscreen OR iPad OR Mobile OR Tablet* OR E?Therap* OR “Information Technology” OR ICT OR “Information communication technolog*” OR “Health Communication” OR “Wireless technolog*” OR Wearable* OR Telecommunication* OR “Information technology device” OR “Communication software” OR “Biomedical software” OR “Healthcare software” OR “Self-care software” OR “Interactive voice response system” OR MAINSUBJECT.EXACT(“Digital technology”) OR MAINSUBJECT.EXACT(“Internet-Based Intervention”) OR MAINSUBJECT.EXACT(“Internet”) OR MAINSUBJECT.EXACT(“Mobile Applications”) OR MAINSUBJECT.EXACT(“Telemedicine”) OR MAINSUBJECT.EXACT(“Multimedia”) OR MAINSUBJECT.EXACT(“Telenursing”) OR MAINSUBJECT.EXACT(“Information Technology”) OR MAINSUBJECT.EXACT(“Wireless Technology”) | 816,419 |
| #4 | #1 AND #2 AND #3 | 94 |

1. **Cochrane Library Search Trail**

| Search # | MeSH Terms and Key Words | Articles No. |
| --- | --- | --- |
| #1 | MeSH descriptor: [Reproductive Techniques] explode all trees OR MeSH descriptor: [Reproductive Techniques, Assisted] explode all trees OR “Reproductive Technic?” OR “Assisted Reproductive Technique?” OR “Assisted Reproductive Technic?” OR “Assisted Reproductive Technolog*” OR “Reproductive Technolog*” OR “Embryo Transfer” OR “Fertility Preservation” OR “Fertilization in Vitro” OR “Insemination, Artificial” OR “Ovulation Induction” OR Superovulation OR “Reproductive procedure” OR “Infertility therapy” OR “Reproductive Technique*” OR “Reproductive Technique*, Assisted” | 10,557 |
| #2 | MeSH descriptor: [Complementary Therapies] explode all trees OR MeSH descriptor: [Acupuncture Therapy] explode all trees OR MeSH descriptor: [Holistic Health] explode all trees OR MeSH descriptor: [Yoga] explode all trees OR MeSH descriptor: [Aromatherapy] explode all trees OR MeSH descriptor: [Counseling] explode all trees OR MeSH descriptor: [Distance Counseling] explode all trees OR MeSH descriptor: [Remote Consultation] explode all trees OR MeSH descriptor: [Cognitive Behavioral Therapy] explode all trees OR MeSH descriptor: [Psychotherapy, Group] explode all trees OR MeSH descriptor: [Nutrition Therapy] explode all trees OR MeSH descriptor: [Dialectical Behavior Therapy] explode all trees OR MeSH descriptor: [Behavioral Disciplines and Activities] explode all trees OR MeSH descriptor: [Rehabilitation] explode all trees OR MeSH descriptor: [Education] explode all trees OR MeSH descriptor: [Self-Help Groups] explode all trees OR MeSH descriptor: [Behavior Therapy] explode all trees OR “Complementary therap*” OR “Complementary Medicine” OR “Alternative Medicine” OR “Acupuncture Therapy” OR Acupotom* OR Anthroposophy OR “Holistic Health” OR “Holistic Therap*” OR “Holistic Health” OR “Mind Body Therap*” OR Aromatherapy OR Psychotherap* OR Meditation OR “Mental Healing” OR “Relaxation Therap*” OR “Therapeutic Touch” OR Yoga OR Aromatherapy OR “Sensory Art Therap*” OR “Art Therap*” OR “Color Therap*” OR “Dance Therap*” OR “Music Therap*” OR “Spiritual Therap*” OR Counceling OR “E Counseling” OR E-Therapy OR “Remote Consultation” OR “Sex Counseling” OR “Cognitive Behavioral Therap*” OR “Cognitive Therap*” OR Psychotherap* OR “diet therapy” OR kinesiotherapy OR Kinesitherapy OR kinesiatrics OR sport OR “Behavior therap*” OR Rehabilitation OR Habilitation OR Education OR “Self Help” OR “Self Help Group” OR “Support Group” OR “Therapeutic Social” OR “Therapeutic Social Club” OR “Behavior Therapies” OR “Behavior Treatment” OR “Conditioning Therap*” OR “Behavior Change Techniq*” OR “Technique, Behavior Change” OR “Behavior Modification” | 328,451 |
| #3 | MeSH descriptor: [Digital Technology] explode all trees OR MeSH descriptor: [Internet-Based Intervention] explode all trees OR MeSH descriptor: [Internet] explode all trees OR MeSH descriptor: [Mobile Applications] explode all trees OR MeSH descriptor: [Telemedicine] explode all trees OR MeSH descriptor: [Multimedia] explode all trees OR MeSH descriptor: [Telenursing] explode all trees OR MeSH descriptor: [Information Technology] explode all trees OR MeSH descriptor: [Wireless Technology] explode all trees OR “Digital health” OR “Digital technolog*” OR “Health technolog*” OR “Internet Based Intervention*” OR “Internet based” OR “Internet intervention*” OR “Web based” OR “Web based intervention*” OR “Computer Assisted” OR “Computer based” OR “Computer mediated” OR “Online intervention*” OR “Online” OR “Mobile App*” OR “Portable Software App*” OR “Software App*” OR “Smartphone App*” OR “Application*” OR “Portable Electronic App*” OR “Electronic App*” OR “Mobile Health” OR “Telemedicine” OR “Tele medicine” OR “mHealth” OR “m health” OR “Remote Consultation” OR “Telenursing” OR Telehealth OR eHealth OR ""e health"" OR Telecare OR Telehealth OR “Assistive technolog*” OR Smart OR Digital OR Virtual OR Media OR Touchscreen OR iPad OR Mobile OR Tablet* OR ""E Therap*"" OR “Information Technology” OR ICT OR “Information communication technolog*” OR “Health Communication” OR “Wireless technolog*” OR Wearable* OR Telecommunication* OR “Information technology device” OR “Communication software” OR “Biomedical software” OR “Healthcare software” OR “Self care software” OR “Interactive voice response system” | 270238 |
| #4 | #1 AND #2 AND #3 | 135 |

1. **Scopus Search Trail**

| Search # | MeSH Terms and Key Words | Articles No. |
| --- | --- | --- |
| #1 | “Reproductive Technic?” OR “Assisted Reproductive Technique?” OR “Assisted Reproductive Technic?” OR “Assisted Reproductive Technolog*” OR “Reproductive Technolog*” OR “Embryo Transfer” OR “Fertility Preservation” OR “Fertilization in Vitro” OR “Insemination, Artificial” OR “Ovulation Induction” OR Superovulation OR “Reproductive procedure” OR “Infertility therapy” | 192043 |
| #2 | “Complementary therap*” OR “Complementary Medicine” OR “Alternative Medicine” OR “Acupuncture Therapy” OR Acupotom* OR Anthroposophy OR “Holistic Health” OR “Holistic Therap*” OR “Holistic Health” OR “Mind Body Therap*” OR Aromatherapy OR Psychotherap* OR Meditation OR “Mental Healing” OR “Relaxation Therap*” OR “Therapeutic Touch” OR Yoga OR Aromatherapy OR “Sensory Art Therap*” OR “Art Therap*” OR “Color Therap*” OR “Dance Therap*” OR “Music Therap*” OR “Spiritual Therap*” OR Counceling OR “E Counseling” OR E-Therapy OR “Remote Consultation” OR “Sex Counseling” OR “Cognitive Behavioral Therap*” OR “Cognitive Therap*” OR Psychotherap* OR “diet therapy” OR kinesiotherapy OR Kinesitherapy OR kinesiatrics OR sport OR “Behavior therap*” OR Rehabilitation OR Habilitation OR Education OR “Group?, Self-Help” OR “Self?Help Group?” OR “Support Group?” OR “Group, Support” OR “Club, Therapeutic Social” OR “Social Club, Therapeutic” OR “Therapeutic Social Club?” OR “Behavior Therapies” OR “Behavior Treatment” OR “Treatment, Behavior” OR “Therapy, Conditioning” OR “Conditioning Therap*” OR “Therapy, Behavior” OR “Behavior Change Techniq*” OR “Technique, Behavior Change” OR “Behavior Modification?” OR “Modification, Behavior" | 10,801,413 |
| #3 | “Digital health” OR “Digital technolog*” OR “Health technolog*” OR “Internet Based Intervention*” OR “Internet-based” OR “Internet intervention*” OR “Web-based” OR “Web?based intervention*” OR “Computer-based” OR “Computer Assisted” OR “Computer based” OR “Computer mediated” OR “Online intervention*” OR “Online” OR “Mobile App*” OR “Portable Software App*” OR “Software App*” OR “Smartphone App*” OR “Application*” OR “Portable Electronic App*” OR “Electronic App*” OR “Mobile Health” OR “Telemedicine” OR “Tele-medicine” OR “mHealth” OR “m-health” OR “Remote Consultation” OR “Telenursing” OR Telehealth OR eHealth OR e-health OR Telecare OR Telehealth OR “Assistive technolog*” OR Smart OR Digital OR Virtual OR Media OR Touchscreen OR iPad OR Mobile OR Tablet* OR E?Therap* OR “Information Technology” OR ICT OR “Information communication technolog*” OR “Health Communication” OR “Wireless technolog*” OR Wearable* OR Telecommunication* OR “Information technology device” OR “Communication software” OR “Biomedical software” OR “Healthcare software” OR “Self-care software” OR “Interactive voice response system” | 38,958,197 |
| #4 | TITLE-ABS-KEY ( "Reproductive Technic?" OR "Assisted Reproductive Technique?" OR "Assisted Reproductive Technic?" OR "Assisted Reproductive Technolog*" OR "Reproductive Technolog*" OR "Embryo Transfer" OR "Fertility Preservation" OR "Fertilization in Vitro" OR "Insemination, Artificial" OR "Ovulation Induction" OR superovulation OR "Reproductive procedure" OR "Infertility therapy" ) AND TITLE-ABS-KEY ( "Complementary therap*" OR "Complementary Medicine" OR "Alternative Medicine" OR "Acupuncture Therapy" OR acupotom* OR anthroposophy OR "Holistic Health" OR "Holistic Therap*" OR "Holistic Health" OR "Mind Body Therap*" OR aromatherapy OR psychotherap* OR meditation OR "Mental Healing" OR "Relaxation Therap*" OR "Therapeutic Touch" OR yoga OR aromatherapy OR "Sensory Art Therap*" OR "Art Therap*" OR "Color Therap*" OR "Dance Therap*" OR "Music Therap*" OR "Spiritual Therap*" OR counceling OR "E Counseling" OR e-therapy OR "Remote Consultation" OR "Sex Counseling" OR "Cognitive Behavioral Therap*" OR "Cognitive Therap*" OR psychotherap* OR "diet therapy" OR kinesiotherapy OR kinesitherapy OR kinesiatrics OR sport OR "Behavior therap*" OR rehabilitation OR habilitation OR education OR "Group?, Self-Help" OR "Self?Help Group?" OR "Support Group?" OR "Group, Support" OR "Club, Therapeutic Social" OR "Social Club, Therapeutic" OR "Therapeutic Social Club?" OR "Behavior Therapies" OR "Behavior Treatment" OR "Treatment, Behavior" OR "Therapy, Conditioning" OR "Conditioning Therap*" OR "Therapy, Behavior" OR "Behavior Change Techniq*" OR "Technique, Behavior Change" OR "Behavior Modification?" OR "Modification, Behavior" ) AND TITLE-ABS-KEY ( "Digital health" OR "Digital technolog*" OR "Health technolog*" OR "Internet Based Intervention*" OR "Internet-based" OR "Internet intervention*" OR "Web-based" OR "Web?based intervention*" OR "Computer-based" OR "Computer Assisted" OR "Computer based" OR "Computer mediated" OR "Online intervention*" OR "Online" OR "Mobile App*" OR "Portable Software App*" OR "Software App*" OR "Smartphone App*" OR "Application*" OR "Portable Electronic App*" OR "Electronic App*" OR "Mobile Health" OR "Telemedicine" OR "Tele-medicine" OR "mHealth" OR "m-health" OR "Remote Consultation" OR "Telenursing" OR telehealth OR ehealth OR e-health OR telecare OR telehealth OR "Assistive technolog*" OR smart OR digital OR virtual OR media OR touchscreen OR ipad OR mobile OR tablet* OR e?therap* OR "Information Technology" OR ict OR "Information communication technolog*" OR "Health Communication" OR "Wireless technolog*" OR wearable* OR telecommunication* OR "Information technology device" OR "Communication software" OR "Biomedical software" OR "Healthcare software" OR "Self-care software" OR "Interactive voice response system" ) AND PUBYEAR > 1999 AND PUBYEAR < 2024 | 335 |

1. **ProQuest Search Trail**

| Search # | MeSH Terms and Key Words | Articles No. |
| --- | --- | --- |
| #1 | “Reproductive Technic?” OR “Assisted Reproductive Technique?” OR “Assisted Reproductive Technic?” OR “Assisted Reproductive Technolog*” OR “Reproductive Technolog*” OR “Embryo Transfer” OR “Fertility Preservation” OR “Fertilization in Vitro” OR “Insemination, Artificial” OR “Ovulation Induction” OR “Superovulation” OR “Reproductive procedure” OR “Infertility therapy” OR “Reproductive Techniques” OR “Reproductive Techniques, Assisted” | 43,299 |
| #2 | “Complementary therap*” OR “Complementary Medicine” OR “Alternative Medicine” OR “Acupuncture Therapy” OR “Acupotom*” OR “Anthroposophy” OR “Holistic Health” OR “Holistic Therap*” OR “Mind-Body Therap*” OR “Aromatherapy” OR “Psychotherap*” OR “Meditation” OR “Mental Healing” OR “Relaxation Therap*” OR “Therapeutic Touch” OR “Yoga” OR “Aromatherapy” OR “Sensory Art Therap*” OR “Art Therap*” OR “Color Therap*” OR “Dance Therap*” OR “Music Therap*” OR “Spiritual Therap*” OR “Counceling” OR “E-Counseling” OR E-Therapy OR “Remote Consultation” OR “Sex Counseling” OR “Cognitive Behavioral Therap*” OR “Cognitive Therap*” OR “Psychotherap*” OR “diet therapy” OR “kinesiotherapy” OR “Kinesitherapy” OR “kinesiatrics” OR “sport” OR “Behavior therap*” OR “Rehabilitation” OR “Habilitation” OR “Education” OR “Group, Self-Help” OR “Self-Help Group?” OR “Support Group?” OR “Group, Support” OR “Club, Therapeutic Social” OR “Social Club, Therapeutic” OR “Therapeutic Social Club” OR “Behavior Therapies” OR “Behavior Treatment” OR “Treatment, Behavior” OR “Therapy, Conditioning” OR “Conditioning Therap*” OR “Therapy, Behavior” OR “Behavior Change Techniq*” OR “Technique, Behavior Change” OR “Behavior Modification” OR “Modification, Behavior" OR “Complementary Therapies” OR “Acupuncture Therapy” OR “Holistic Health” OR “Yoga” OR “Aromatherapy” OR “Counseling” OR “Distance Counseling” OR “Remote Consultation” OR “Cognitive Behavioral Therapy” OR “Cognitive therapy” OR “Psychotherapy, group” OR “Nutrition Therapy” OR “Behavior therapy” OR “Behavioral Disciplines and Activities” OR “Rehabilitation” OR “Education” OR “Self-help groups” OR “Behavior therapy” | 2,407,946 |
| #3 | "Digital health" OR "Digital technolog*" OR "Health technolog*" OR "Internet Based Intervention*" OR "Internet-based" OR "Internet intervention*" OR "Web-based" OR "Web-based intervention*" OR "Computer-based" OR "Computer Assisted" OR "Computer based" OR "Computer mediated" OR "Online intervention*" OR "Online" OR "Mobile App*" OR "Portable Software App*" OR "Software App*" OR "Smartphone App*" OR "Application*" OR "Portable Electronic App*" OR "Electronic App*" OR "Mobile Health" OR "Telemedicine" OR "Tele-medicine" OR "mHealth" OR "m-health" OR "Remote Consultation" OR "Telenursing" OR “Telehealth” OR “eHealth” OR “e-health” OR “Telecare” OR “Telehealth” OR "Assistive technolog*" OR “Smart” OR “Digital” OR “Virtual” OR “Media” OR “Touchscreen” OR “iPad” OR “Mobile” OR “Tablet*” OR “E-Therap*” OR "Information Technology" OR “ICT” OR "Information communication technolog*" OR "Health Communication" OR "Wireless technolog*" OR “Wearable*” OR “Telecommunication*” OR "Information technology device" OR "Communication software" OR "Biomedical software" OR "Healthcare software" OR "Self-care software" OR "Interactive voice response system" OR “Internet" OR "Mobile Applications" OR “Telemedicine" OR "Multimedia" OR "Telenursing" OR "Information Technology" OR "Wireless Technology" | 6,116,424 |
| #4 | #1 AND #2 AND #3 | 327 |

1. **Ovid-Medline Search Trail**

| Search # | MeSH Terms and Key Words | Articles No. |
| --- | --- | --- |
| #1 | ("Reproductive Technic?" or "Assisted Reproductive Technique?" or "Assisted Reproductive Technic?" or "Assisted Reproductive Technolog$" or "Reproductive Technolog$" or "Embryo Transfer" or "Fertility Preservation" or "Fertilization in Vitro" or "Insemination, Artificial" or "Ovulation Induction" or Superovulation or "Reproductive procedure" or "Infertility therapy").ti,ab. or exp Reproductive Techniques/ or exp Reproductive Techniques, Assisted/ | 174,636 |
| #2 | (“Complementary therap$” OR “Complementary Medicine” OR “Alternative Medicine” OR “Acupuncture Therapy” OR Acupotom$ OR Anthroposophy OR “Holistic Health” OR “Holistic Therap$” OR “Mind Body Therap$” OR Aromatherapy OR Psychotherap$ OR Meditation OR “Mental Healing” OR “Relaxation Therap$” OR “Therapeutic Touch” OR Yoga OR Aromatherapy OR “Sensory Art Therap$” OR “Art Therap$” OR “Color Therap$” OR “Dance Therap$” OR “Music Therap$” OR “Spiritual Therap$” OR Counceling OR “E Counseling” OR E-Therapy OR “Remote Consultation” OR “Sex Counseling” OR “Cognitive Behavioral Therap$” OR “Cognitive Therap$” OR Psychotherap$ OR “diet therapy” OR kinesiotherapy OR Kinesitherapy OR kinesiatrics OR sport OR “Behavior therap$” OR Rehabilitation OR Habilitation OR Education OR “Group, Self-Help” OR “Self Help Group?” OR “Support Group?” OR “Group, Support” OR “Club?”, “Therapeutic Social” OR “Social Club?”, Therapeutic OR “Therapeutic Social Club?” OR “Behavior Therapies” OR “Behavior Treatment” OR “Treatment, Behavior” OR “Therapy, Conditioning” OR “Conditioning Therap$” OR “Therapy, Behavior” OR “Behavior Change Techniq$” OR “Technique, Behavior Change” OR “Behavior Modification?” OR “Modification, Behavior").ti,ab. OR EXP Complementary Therapies/ OR EXP Acupuncture Therapy/ OR EXP Holistic Health/ OR EXP Yoga/ OR EXP Aromatherapy/ OR EXP Counseling/ OR EXP Distance Counseling/ OR EXP Remote Consultation/ OR EXP Cognitive Behavioral Therapy/ OR EXP Cognitive therapy/ OR EXP Psychotherapy, group/ OR EXP Nutrition Therapy/ OR EXP Behavior therapy/ OR EXP “Behavioral Disciplines and Activities”/ OR EXP Rehabilitation/ OR EXP Education/ OR EXP Self-help groups/ OR EXP Behavior therapy/ | 2,745,760 |
| #3 | (“Digital health” OR “Digital technolog$” OR “Health technolog$” OR “Internet Based Intervention$” OR “Internet-based” OR “Internet intervention$” OR “Web-based” OR “Web based intervention$” OR “Computer-based” OR “Computer Assisted” OR “Computer based” OR “Computer mediated” OR “Online intervention$” OR “Online” OR “Mobile App$” OR “Portable Software App$” OR “Software App$” OR “Smartphone App$” OR “Application$” OR “Portable Electronic App$” OR “Electronic App$” OR “Mobile Health” OR “Telemedicine” OR “Tele-medicine” OR “mHealth” OR “m-health” OR “Remote Consultation” OR “Telenursing” OR Telehealth OR eHealth OR e-health OR Telecare OR Telehealth OR “Assistive technolog$” OR Smart OR Digital OR Virtual OR Media OR Touchscreen OR iPad OR Mobile OR Tablet$ OR E Therap$ OR “Information Technology” OR ICT OR “Information communication technolog$” OR “Health Communication” OR “Wireless technolog$” OR Wearable$ OR Telecommunication$ OR “Information technology device” OR “Communication software” OR “Biomedical software” OR “Healthcare software” OR “Self-care software” OR “Interactive voice response system”).ti,ab. OR exp Digital technology/ OR exp Internet-Based Intervention/ OR exp Internet/ OR exp Mobile Applications/ OR exp Telemedicine/ OR exp Multimedia/ OR exp Telenursing/ OR exp Information Technology/ OR exp Wireless Technology/ | 2,028,201 |
| #4 | #1 AND #2 AND #3 AND yr"2000 - 2023" | 780 |
